# Alterations in neurovascular coupling following acute traumatic brain injury

**DOI:** 10.1117/1.NPh.4.4.045007

**Published:** 2017-12-23

**Authors:** Hyounguk Jang, Stanley Huang, Daniel X. Hammer, Lin Wang, Harmain Rafi, Meijun Ye, Cristin G. Welle, Jonathan A. N. Fisher

**Affiliations:** a New York Medical College, Department of Physiology, Valhalla, New York, United States; b U.S. Food and Drug Administration, Division of Biomedical Physics, Silver Spring, Maryland, United States; c University of Colorado Denver, Departments of Neurosurgery and Bioengineering, Aurora, Colorado, United States

**Keywords:** brain injury, diffuse correlation spectroscopy, neurophotonics, neurophysiology

## Abstract

Following acute traumatic brain injury (TBI), timely transport to a hospital can significantly improve the prognosis for recovery. There is, however, a dearth of quantitative biomarkers for brain injury that can be rapidly acquired and interpreted in active, field environments in which TBIs are frequently incurred. We explored potential functional indicators for TBI that can be noninvasively obtained through portable detection modalities, namely optical and electrophysiological approaches. By combining diffuse correlation spectroscopy with colocalized electrophysiological measurements in a mouse model of TBI, we observed concomitant alterations in sensory-evoked cerebral blood flow (CBF) and electrical potentials following controlled cortical impact. Injury acutely reduced the peak amplitude of both electrophysiological and CBF responses, which mostly recovered to baseline values within 30 min, and intertrial variability for these parameters was also acutely altered. Notably, the postinjury dynamics of the CBF overshoot and undershoot amplitudes differed significantly; whereas the amplitude of the initial peak of stimulus-evoked CBF recovered relatively rapidly, the ensuing undershoot did not appear to recover within 30 min of injury. Additionally, acute injury induced apparent low-frequency oscillatory behavior in CBF (<1  Hz). Histological assessment indicated that these physiological alterations were not associated with any major, persisting anatomical changes. Several time-domain features of the blood flow and electrophysiological responses showed strong correlations in recovery kinetics. Overall, our results reveal an array of stereotyped, injury-induced alterations in electrophysiological and hemodynamic responses that can be rapidly obtained using a combination of portable detection techniques.

## 1 Introduction

In the United States, each year there are over 1.5 million traumatic brain injuries (TBIs), resulting in 50,000 deaths.^1^ TBI induces temporary or permanent impairment of cognition, physical function, and psychosocial behavior.^2^ TBI severity ranges widely depending on the nature of the injury. The majority of these injuries are classified as “mild” TBI (mTBI), which are challenging to diagnose and track because quantitative biomarkers for mTBI are lacking. In fact, one of the defining characteristics of mTBI is that it cannot be validated through standard methods of clinical imaging.^3^ Given the difficulty of rapid detection, mTBI poses a particular challenge to public health because repeated injuries such as concussions have a cumulative effect on brain health.^4^^–^^6^

Among a wide spectrum of TBI sequelae, sensory and cognitive deficits are some of the most common.^7^ A difficulty in assessing these deficits is that they are frequently mutually confounding. For example, auditory dysfunction following injury includes both peripheral deficits, e.g., increased hearing thresholds, as well as difficulties with more sophisticated auditory tasks, such as discriminating sounds in noisy environments or comprehending speech.^8^ Visual deficits following TBI are similarly complex; the most common complaints, for example, are accommodative deficiencies,^9^ which include blurred vision, headache, motion sickness, or loss of concentration during visual task performance.^10^ While auditory and visual performance may be difficult to dissociate from cognitive deficits, olfaction, which does not involve significant feedback with subcortical structures, is also impacted by TBI, and the degree of anosmia is correlated with the severity of injury.^11^

The integrity of sensory systems can be probed electrophysiologically via evoked potentials (EPs). EPs can be measured noninvasively and comprise a series of positive and negative voltage deflections, which reflect the afferent relay and processing of sensory information. Auditory-, visual-, and somatosensory-evoked potentials (SSEPs) are routinely used in multiple clinical contexts to aid neurological assessment of TBI and to monitor functional recovery over time.^12^ Sensory-evoked neural activity can also be inferred based on cerebral hemodynamic responses, including changes in cerebral blood flow (ΔCBF), volume, and oxygenation that are evoked by brief sensory stimuli. Damage to any portion of the cerebral vasculature fundamentally alters the ability of the network to supply neurons with energy.^13^ In fact, blockage of even single capillaries can cause larger-scale changes in blood flow^14^ and may result in microvascular ischemia.^15^ Repercussions of these injuries, even if mild, may continue to progress following the primary trauma, ultimately leading to more global sequelae.

We explored the possibility of deriving innovative indicators for mTBI based on intrinsic correlations between hemodynamic and neuronal activity. Specifically, we investigated signals that can be measured rapidly and using portable technology, which is a prerequisite for expedited assessment. To noninvasively monitor sensory-evoked hemodynamics, we used diffuse correlation spectroscopy (DCS) that takes advantage of the dynamic scattering properties of red blood cells to directly measure cerebral blood flow (CBF). DCS is particularly sensitive to flow in the cortical microvasculature due to the high absorption (and thus low probability of photon escape) in larger blood vessels.^16^ DCS has been used to measure functional hemodynamics associated with sensory stimuli and motor tasks^17^^,^^18^ and has been used to track baseline CBF following brain trauma.^19^ We supplemented our ongoing optical recordings of the sensory-evoked ΔCBF with concomitant measurements of SSEPs and applied this multimodal approach to a mouse model of TBI that employed controlled cortical impact (CCI) as the source of primary injury.^20^^–^^22^ The ability to noninvasively monitor both aspects of neural response enabled us to obtain the first detailed, *in vivo* portrait of the effects of acute injury on sensory processing in the brain.

## 2 Materials and Methods

### 2.1 Surgical Procedures

**Fig. 1:**
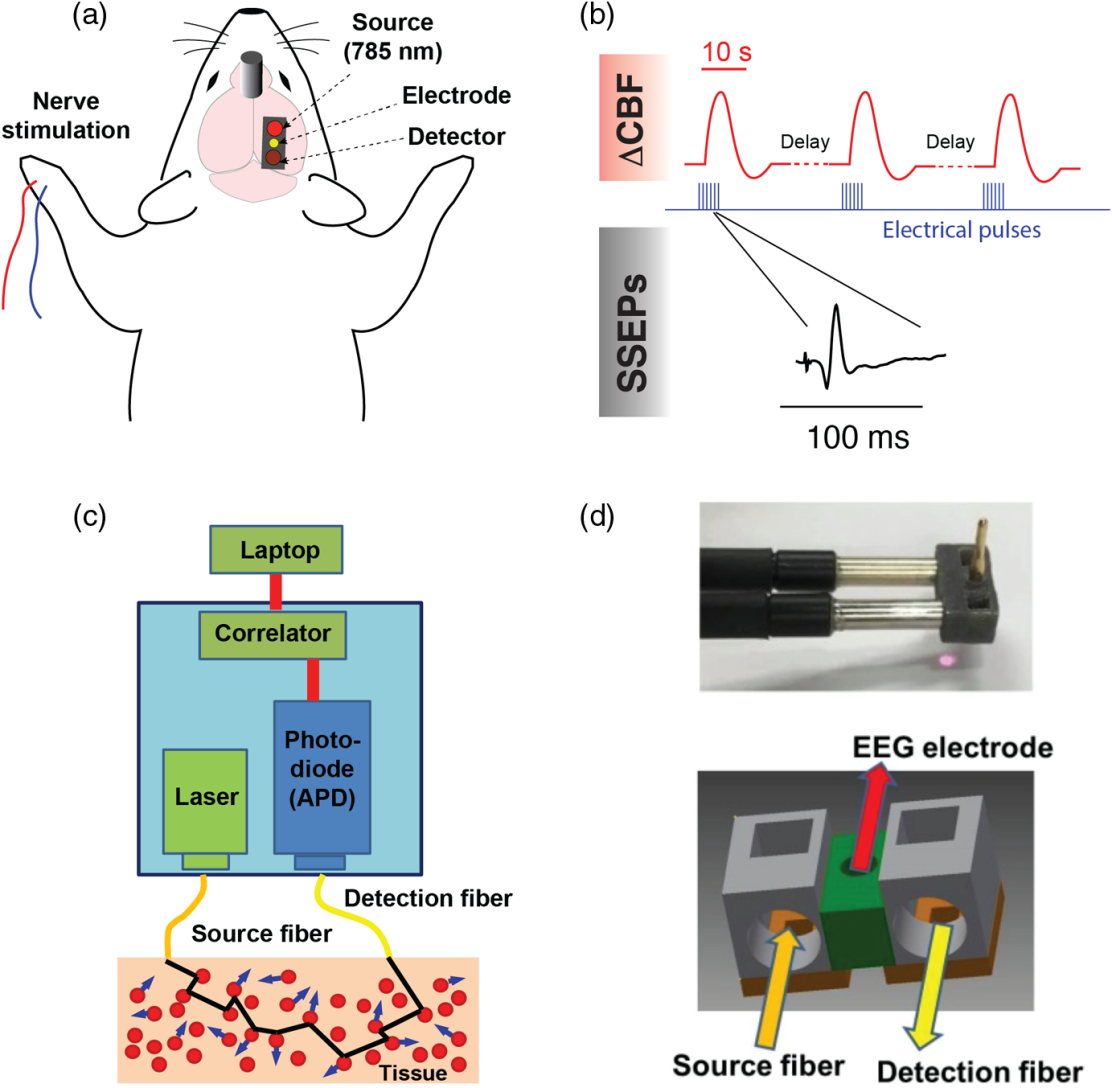
Experimental design and measurement apparatus. (a) Schematic of the recording and stimulation configuration. A DCS probe tip containing optical fibers is placed on the region of the skull above the primary somatosensory cortex, at a location coinciding with the forepaw’s representation. A recording EEG electrode is inserted through the center of the probe. Pulses of electrical current are delivered to the contralateral limb with respect to the optical and electrophysiological measurements. Closed-skull impact is delivered by a 5-mm impactor tip ∼4-mm anterior and ∼1-mm lateral of bregma contralateral to the optical measurement (see Sec. 2). (b) Overview of synchronization between stimulus, measurements of CBF, and SSEPs. The red traces depict simulated hemodynamic signals, which are broad compared with electrophysiological potentials (note the time scale difference in the insets). The blue pulses indicate electrical stimuli delivered to the median nerve. (c) Block diagram of the DCS device used in this study. (d) Photograph and schematic of the 3-D-printed DCS probe tip. Light is delivered and collected from the tissue through fiber optics that are coupled to the skin with microprisms.

All animal experiments were performed in accordance with the guidelines of the White Oak Institutional Animal Care and Use Committee. Optical and electrophysiological measurements were performed on 10 male C57BL/6J mice (12 to 24 weeks). Anesthesia was induced by an initial exposure to 4% isoflurane (vaporized in medical grade compressed oxygen) for <30  s. Animals were additionally administered an injection of xylazine (18  mg/kg, IP) to provide a stable plane of anesthesia at low isoflurane concentrations (0.1% to 0.25%) for the remainder of the experimental session, which typically lasted 3 to 6 h. Maintenance doses of xylazine (6  mg/kg) were administered once every ∼2.5  h. Following initial anesthesia induction, animals were positioned in a stereotaxic apparatus (David Kopf Instruments, California). Their body temperature was measured and maintained at 37°C with a closed-loop temperature-controlled heating pad (Model TC-1000, CWE). Respiratory rate was also monitored and maintained at ∼100  breaths/min during the surgical and experimental procedures. Skin incisions were infused with lidocaine, and the eyes were covered with ointment (Lacri-Lube) to prevent drying. A midline sagittal incision was made in the skin, which exposed the coronal and lambdoid sutures on the skull. The intersection of these sutures with the midline (i.e., bregma and lambda) served as landmarks for recording locations and was also used as a guide when drilling burr holes. The area of the skull under the probe was cleaned with 70% ethanol, and the optical probe was secured to the skull by cyanoacrylate glue (Loctite 454, Hankel, Australia) [Fig. 1(a)]. Burr holes (diameter: ∼0.5  mm) were made for placement of two silver wire electrodes: a recording electrode, placed 2.5-mm lateral and 1-mm posterior to bregma, and a reference electrode, placed 1-mm lateral and 1-mm posterior to lambda. The recording electrode was embedded within the optical probe tip, and the reference electrode was secured into position with Kwik-Sil adhesive (World Precision Instruments, Florida). A ground needle electrode was placed subcutaneously on the back of the animal.

Although epidural measurements of SSEPs are invasive, we have previously compared the effects of injury on SSEPs measured both epidurally and epidermally.^23^^,^^24^ In mouse experiments, SSEP waveforms measured using both approaches differ only in signal-to-noise ratio, which is roughly an order of magnitude higher when measured epidurally. In this study, the use of epidural recordings permitted us to rapidly acquire clear SSEPs at a high sampling rate following injury owing to the fact that less time was needed to be spent averaging, compared with epidermal recordings. This enhanced the temporal resolution with which injury-induced changes could be tracked.

### 2.2 Sensory Stimuli and Electrophysiological Recordings

SSEPs were recorded in single-ended configuration (RZ5D processor, PZ2 preamplifier, ZC16 headstage, Tucker-Davis Technologies, Florida) with a shared common reference electrode, at a sampling frequency of 3 kHz. Optical measurements of ΔCBF were performed concurrently, driven by a separate computer. The median nerve contralateral to the recording locations was stimulated via a pair of 27-gauge stainless-steel needles inserted subcutaneously into the forelimb of the animal. The electrical stimulus consisted of a train of 12 current pulses generated by a constant current stimulator (DS7A, Digitimer) (amplitude 4 mA, frequency 3 Hz, pulse duration 200  μs, and total pulse-train duration 4 s). Individual measurements of sensory-evoked ΔCBF were separated by 45 s, which we empirically found to be the minimum intertrial duration that did not elicit alterations in the steady-state CBF. An overview of the acquisition and stimulus timing is shown in Fig. 1(b). The major peaks associated with the SSEP waveform were identified with MATLAB^®^ as maxima and minima within boundaries set using published values as a reference ^23^^,^^25^^,^^26^ as follows: P1 corresponded to the time of maximum voltage within the window of 15 to 30 ms, N1 corresponded to the time of minimum voltage within the window of 21 to 50 ms, and P2 corresponded to the time of maximum voltage at times later than the identified N1 time.

### 2.3 Controlled Cortical Impact

CCI is a well-established and highly reproducible brain injury model.^20^^,^^21^^,^^27^^–^^30^ Closed-skull impact was delivered with an Impact One™ stereotaxic impactor (Leica Microsystems). Using a 5-mm-diameter metal impact tip, we used the following settings: velocity 4  m/s, dwell time 100 ms, and impact depth 0.6 mm. Strike velocities >5  m/s and/or strike depths>0.8  mm resulted in skull fracture. Additionally, impactor tips with smaller diameter tended to cause fractures. The metal impactor tip was positioned over the exposed skull ∼4-mm anterior and ∼1-mm lateral of bregma, contralateral to the optical measurement.

### 2.4 Optical Measurement of Cerebral Blood Flow

The DCS signal can be used to measure blood flow by using speckle correlation techniques. The basic theory underlying DCS has been extensively described in previous publications.^16^^,^^31^ Briefly, when laser light migrates through tissue, the emerging intensity pattern, called a speckle pattern, is composed of bright and dark spots, which are caused by constructive and destructive interference of photons that traverse different path lengths. The intensity fluctuations of a single region, which are caused by interactions of scattered light with moving particles (i.e., red blood cells), can be utilized to extract information about blood flow.

A block diagram of our DCS recording system is shown in Fig. 1(c). It consists of a long coherence length continuous-wave near-infrared laser (785 nm, CrystaLaser, Nevada), a photon-counting avalanche photodiode (APD) (SPCM-AQRH-12-FC, Excelitas, Quebec, Canada), and an autocorrelator signal processing board (Ref. 32, New Jersey). NIR excitation light was delivered to the brain with a multimode optical fiber (200-μm core diameter, Thorlabs, New Jersey), and the scattered light was detected with a single-mode fiber (5-μm core diameter) connected to the APD. The source and detection fibers were separated by 5 mm. The APD signal was sent to the correlator board, which computed the intensity of the autocorrelation function. The correlation board streams output signals continuously via USB to a laptop PC for further data analysis.

The probe that coupled the optical fibers and recording electrode to the head was fabricated from semiflexible acrylate polymer using a three-dimensional (3-D) printer (Objet 260 Connex 3 printer, Stratasys, Minnesota). Embedded microprisms directed light from the fibers down to the head. The recording electrode was situated at the midpoint between the DCS optical source–detector separation. The midpoint of the probe [indicated in green in Fig. 1(d)] was thinner and permitted the probe to bend and conform to the curvature of the mouse’s skull, providing tight contact to the surface. The probe was positioned on a relatively flat region of intact skull above the forelimb’s representation in primary somatosensory cortex, 2.5-mm lateral of the midline at the same coordinate as bregma on the rostrocaudal axis.^33^

### 2.5 Modeling the Hemodynamic Response Function

**Fig. 2:**
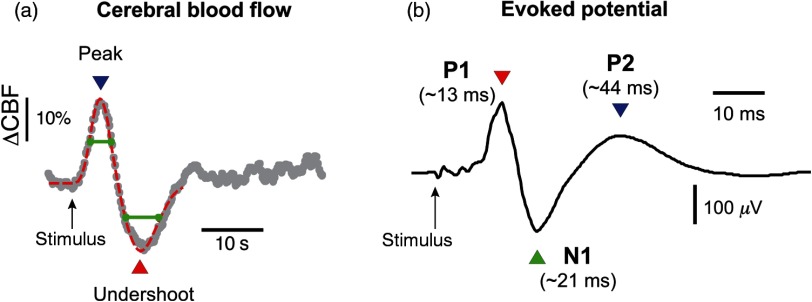
Somatosensory-evoked hemodynamic and electrophysiological responses. (a) Optically measured changes in relative CBF following electrical stimulation of the median nerve. The gray trace represents the average of 10 trials in one experiment; blood flow values are normalized to the average of CBF values recorded in 4 s prior to the stimulus onset, which is indicated by the arrow. The dotted red line that is superimposed on the trace represents a fit to the observed hemodynamic response using a conventional HRF model (two-gamma functions), as described in Sec. 2. (b) SSEP recorded concurrently during acquisition of the optical data depicted in (a). The prominent peaks P1, N1, and P2 occur at temporal latencies that are well-established in the literature. The trace represents the average obtained from the same 10 trials in (a); however, for each trial, there are 12 SSEPs recorded in response to individual stimulus pulses (4 s of pulses delivered at 3 Hz); thus, the trace represents the average of 120 SSEPs. The green lines represent the widths of positive and negative peaks, measured at half of the maximum/minimum values. Note that the timescale in (a) is roughly 1000× longer than in (b).

To extract quantitative features from the sensory-evoked ΔCBF, we fit the observed waveform to a canonical hemodynamic response function (HRF) that uses two-gamma density functions to approximate the hemodynamic response [Fig. 2(a)],^34^ i.e.,

$$\mathrm{HRF}(t)=A[\frac{t^{\alpha_{1}-1}\beta_{1}^{\alpha_{1}}e^{-\beta_{1}t}}{\Gamma (\alpha_{1})}-c\frac{t^{\alpha_{2}-1}\beta_{2}^{\alpha_{2}}e^{-\beta_{2}t}}{\Gamma (\alpha_{2})}],$$

where Γ represents the gamma function. A, c, $\alpha_{1}$, $\alpha_{2}$, $\beta_{1}$, and $\beta_{2}$ are the fitting coefficients, which were fit using the Levenberg–Marquardt algorithm in MATLAB^®^. Here, A controls the amplitude, c determines the ratio of the response to undershoot, and $\alpha_{1}$, $\alpha_{2}$, $\beta_{1}$, and $\beta_{2}$ control the shape of the fit function. We obtained four major features, which are the amplitude and temporal delay (relative to stimulus onset) of the ΔCBF onset (initial peak) and offset (return to baseline), to quantitatively describe the blood flow response based on the fitted curve. Although this model is typically applied to aspects of the hemodynamic response other than ΔCBF, namely the blood-oxygen-level-dependent signal, the model is generic in that it can accommodate a bimodal onset/offset function. For instance, the model can just as easily fit a response with no apparent undershoot and can capture waveform attributes such as a highly skewed peak. For quantifying trends in the CBF waveforms, the peak and undershoot were identified based on the fit to the dual gamma distribution model, and a criterion of $R^{2}>0.9$ was used in the fitting process.

### 2.6 Data Quality Criteria for Inclusion

Animals varied in the magnitude of noise associated with CBF and electrophysiological measurements. Sources of variability included optical coupling efficiency, electrode impedances, and physiological noise, among others. To best quantify injury-related changes, we only included data from experiments in which preinjury optical and electrophysiological noise levels were low enough to permit the observation of clear stimulus-evoked CBF changes and SSEPs. Quantitatively, our data inclusion criterion for optical measurements was that the initial CBF peak had an amplitude that exceeded twice the standard deviation (SD) of the baseline signal (i.e., z-score>2).

### 2.7 Time-Frequency Analysis of the Hemodynamic Response Function

In the interest of parsing apparent oscillatory behavior associated with postinjury modifications in the hemodynamic response following the main positive and negative peaks, we performed spectrotemporal analysis on the CBF data throughout the entire duration of six experiments. Briefly, the continuous DCS data were divided into six 5-min epochs, and analysis of the evoked responses within those epochs was used to inform single “frames.” We utilized time-frequency transform functions from the MATLAB^®^ toolbox EEGLAB^35^ to produce spectrograms that quantified power as well as intertrial coherence (ITC), which indicates the degree of phase locking in the observed ΔCBF. Note that for each data point in Figs. 9 and 10, the spectrograms were based on only the trials within the 5-min epochs; although a larger sample size generally improves signal-to-noise, in this case because the ΔCBF was dynamically changing in time following injury, there was a trade-off between signal-to-noise and the temporal resolution with which the time evolution of power and ITC could be quantified. We empirically converged on 5-min epochs as a compromise based on observations from the continuous CBF that indicated that recovery generally occurred on a timescale slower than 5 min following a rapid decrement due to CCI.

### 2.8 Histological Procedures

We assessed the effects of CCI on the brain by performing vital staining and immunohistological analysis. Two mice were subjected to CCI using the same parameters as all of the functional monitoring experiments (velocity: 4  m/s; dwell time: 100 ms; and impact depth: 0.6 mm). Another two animals were subjected to CCI of higher severity (impact depth was increased to 1.1 mm), and an additional two sham animals received no impact yet were positioned in the stereotaxic apparatus and the cortical impactor was fixed above the animal’s head. Animals were permitted to recover following the procedure. 24 h after CCI or sham treatment, animals were anesthetized with sodium pentobarbital (100  mg/kg, i.p.) and perfused transcardially with saline and then formalin. Mice were then decapitated and the brain removed and postfixed in formalin overnight. 50-μm coronal brain sections were acquired with a vibrating microtome at anteroposterior (AP) levels spanning bregma +2  mm through −2  mm. For glial fibrillary acidic protein (GFAP) immunohistological analysis, floating slices were incubated overnight with monoclonal antimouse GFAP antibody (1:500 dilution, Invitrogen). The slices were then incubated with Peroxidase-conjugated AffiniPure Goat Antirat IgG (H+L) antibody (1:100 dilution, Jackson ImmunoResearch Laboratories) and stained with a 3,3-diaminobenzidine horseradish peroxidase substrate kit (Vector Laboratories), then mounted onto gelatin subbed slides and coverslipped. For Nissl staining, sections from the same brain, yet AP +50  μm to the locations at which slices for GFAP analysis, were mounted and dried on gelatin subbed slides. They were then immersed in a heated solution of 0.1% cresyl violet (CV) acetate (Electron Microscopy Sciences) for 4 min and dehydrated in alcohol. Slides were then cover slipped with Permount (Fisher Scientific) and dried before imaging.

## 3 Results

### 3.1 Concurrent Measurements of Sensory-Evoked Cerebral Hemodynamics and SSEPs

Using the DCS component of our multimodal apparatus, we recorded sensory-evoked changes in CBF concurrently with electrophysiological measurements of SSEPs. Figure 2(a) shows an example of the preinjury CBF response. Averaged over 10 animals, the ΔCBF displayed an initial increase in blood flow of 14%±4.8% (mean±SD) that peaked at 9±0.3  s, followed by a subsequent undershoot of magnitude −12%±2.5%, which reached a negative peak 17±1.1  s after the stimulus (12±1.1  s after the first peak). CBF returned to baseline values roughly 10 s following maximum undershoot. In terms of electrophysiological recordings, SSEPs displayed well-defined peaks, most prominently a positive deflection (P1) at ∼12-ms poststimulus, a negative peak (N1) at ∼21  ms, and a broader positive peak at ∼44  ms (P2) [Fig. 2(b)].

### 3.2 TBI Acutely Alters Hemodynamic and Electrophysiological Functional Responses

**Fig. 3:**
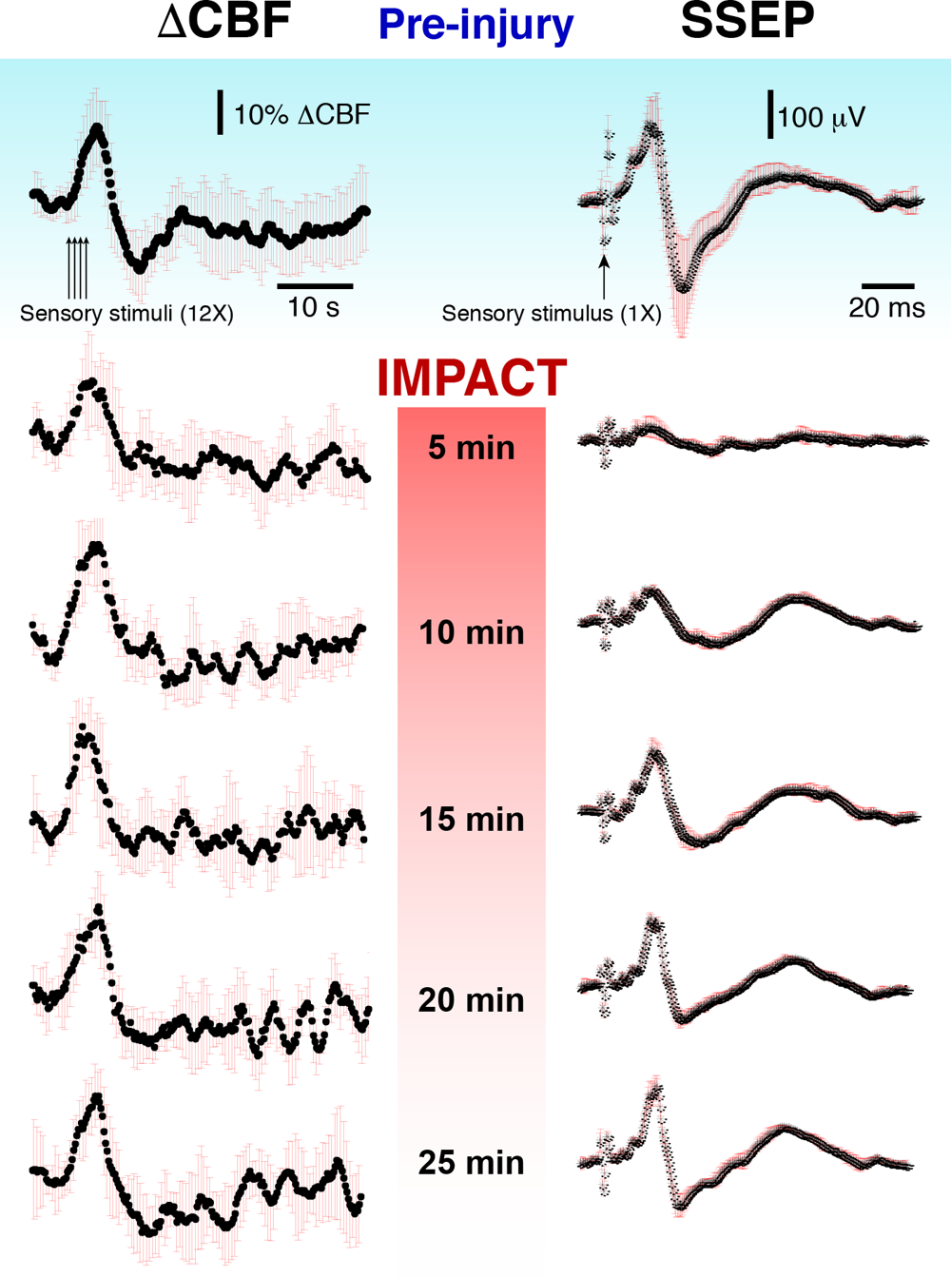
Sensory-evoked blood flow responses from forepaw stimulation for a representative experiment pre- and postinjury. The DCBF and SSEP traces shown here represent the average of multiple stimulations (average of 10 trials preinjury and 5 trials for each time point postinjury). The black line is the average, and the red error bars represent a SD.

**Fig. 4:**
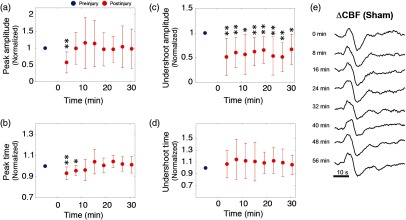
(a)–(d) The grand average summary of the effects of CCI on the amplitude and latency of prominent time-domain features of the ΔCBF waveform. The red dots represent the average values over all animals (n=10). Values are normalized to the baseline value (blue dot). The time of impact is designated here as t=0  s. Error bars represent SD. Significant changes relative to baseline are indicated by asterisks, which refer to P-values obtained from a two-tailed, paired student’s t-test (*=P<0.05, **=P<0.01). (e) A montage of sensory-evoked ΔCBF waveforms taken repeatedly over the course of an hour in a sham experiment.

Cortical impact acutely reduced the amplitude and altered the waveforms of sensory-evoked hemodynamic and electrophysiological responses (Fig. 3). Within the first 5 min following impact, the ΔCBF response peak decreased by ∼50%, and the undershoot amplitude decreased as well, though to a lesser extent (∼20% to 40%). The peak latencies additionally displayed changes on the order of ∼10%, though with opposite trends; the initial increase in CBF peaked ∼1  s earlier than preinjury, and the undershoot peaked ∼1  s later than in the preinjury case. The alterations in these parameters, based on the results of 10 mice, are summarized in Fig. 4. The peak-to-peak amplitude recovered to ∼80% of its baseline value within 30 min, and during this period, the shifts in peak latencies resolved completely. It should be noted that despite being more sensitive to injury than other features of the ΔCBF waveform, the initial peak also recovered at a relatively fast rate, and within 5 to 10 min following injury, the peak amplitude had essentially recovered completely. In contrast, the undershoot alterations following injury, though more difficult to quantify because of the variability in that portion of the waveform post-CCI, displayed no recovery trend within the same time period.

**Fig. 5:**
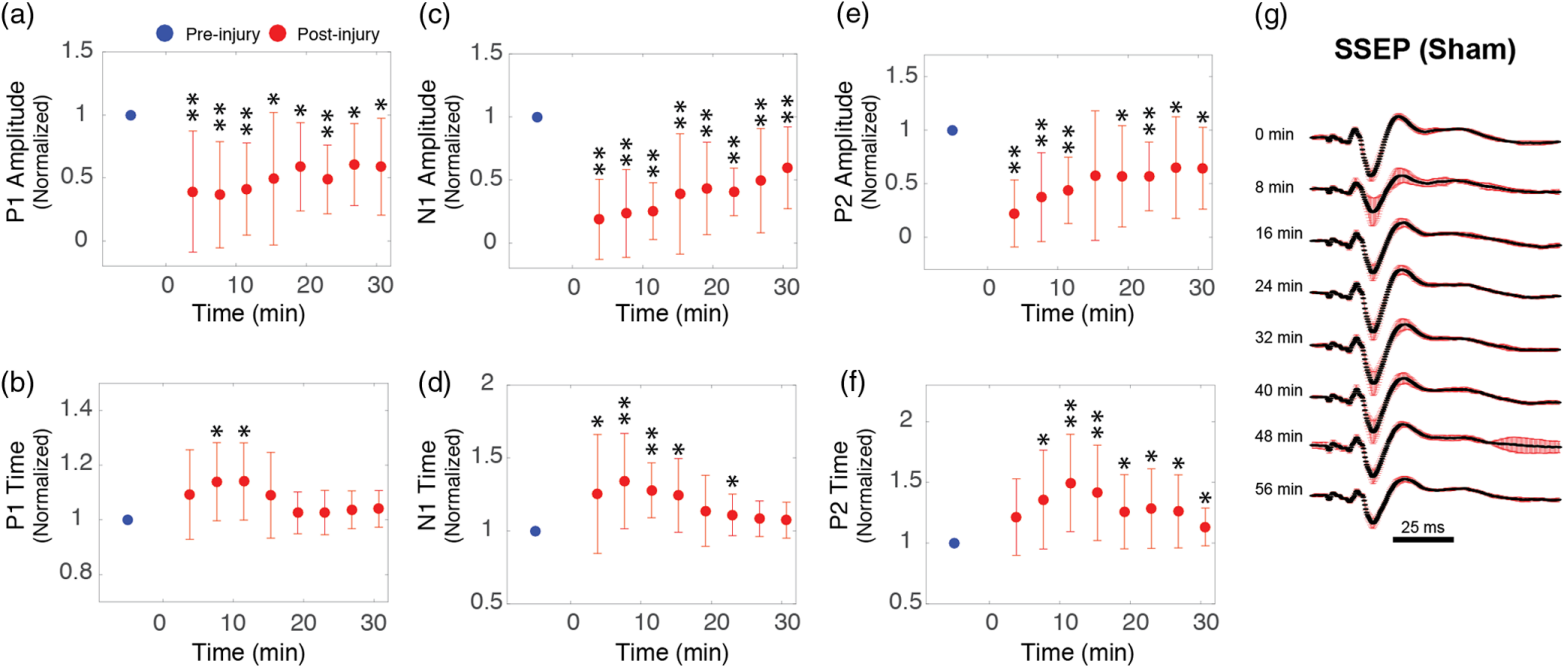
(a)–(f) The grand average summary of the effects of CCI on major SSEP peaks. As in Fig. 4, red data points indicate postinjury averages over all animals (n=10)±SD and are normalized to baseline values (blue data points). The time of impact is designated here as t=0  s, and significance relative to baseline is indicated by asterisks, as in Fig. 4. (g) A montage of SSEP waveforms taken repeatedly over the course of an hour in a sham experiment.

SSEPs displayed similarly profound alterations within the first 5 min after CCI. As shown in Fig. 5, P1 fell to ∼40% of its preinjury amplitude; however, N1 and P2 exhibited greater reductions of 81% and 78%, respectively. CCI additionally altered the peak latencies. Although the P1 latency increased by less than ∼14%, N1 and P2 both increased by up to ∼13% and ∼50%. In general, P1 appeared to be the least sensitive to injury, in terms of both amplitude and latency. In the ensuing 30 min, the peak latencies and amplitudes largely—although not entirely—returned to their baseline values, yet at differing rates. The amplitude of P2, for example, recovered more rapidly than N1 or P1 in the first 15 min. Overall, however, following CCI, the initial decrease in SSEP amplitude (peak-to-peak, N1 to P2) was significantly more profound than the drop in CBF. For example, SSEP peak-to-peak amplitude was reduced by nearly 75%, while CBF peak-to-peak dropped by only ∼45%.

**Fig. 6:**
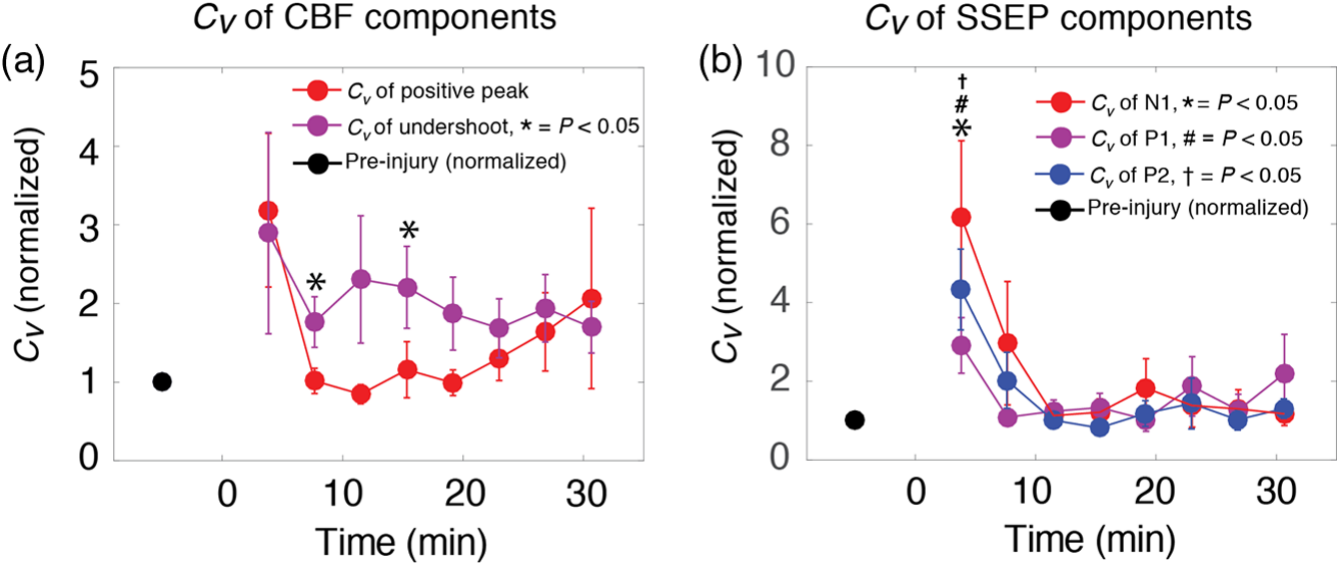
Alterations in the variability of evoked responses following injury. (a) Dynamics of the ΔCBF variability are plotted over time, normalized to a baseline value (black dot). Injury was induced at 0 min in these plots. Variability is quantified here in terms of the coefficient of variation, $C_{v}$, defined as the ratio of the SD to the mean. $C_{v}$ is plotted for both the initial ΔCBF peak (in red) and the subsequent undershoot (purple). (b) Evolution of $C_{v}$ for SSEP peaks N1 (red), P1 (purple), and P2 (blue), normalized to baseline values (black dot). Error bars represent standard error of the mean, and P-value indications are as described in the figure insets.

In addition to alterations in average amplitude and latency of SSEP and CBF features, injury acutely increased their trial-to-trial variability of these parameters (Fig. 6). In our time-domain measurements, we quantified this variability in terms of the coefficient of variation, $C_{v}$, which is defined as the ratio of the SD to the mean value. In the first 5 min following CCI, $C_{v}$ increased by over 200% for all electrophysiological and blood flow parameters. Additionally, while the variability of other parameters returned to near baseline values within 10 min after injury, the CBF undershoot amplitude maintained an elevated variability over 30 min after injury, on average.

### 3.3 Histological Assessment of the Effects of CCI

**Fig. 7:**
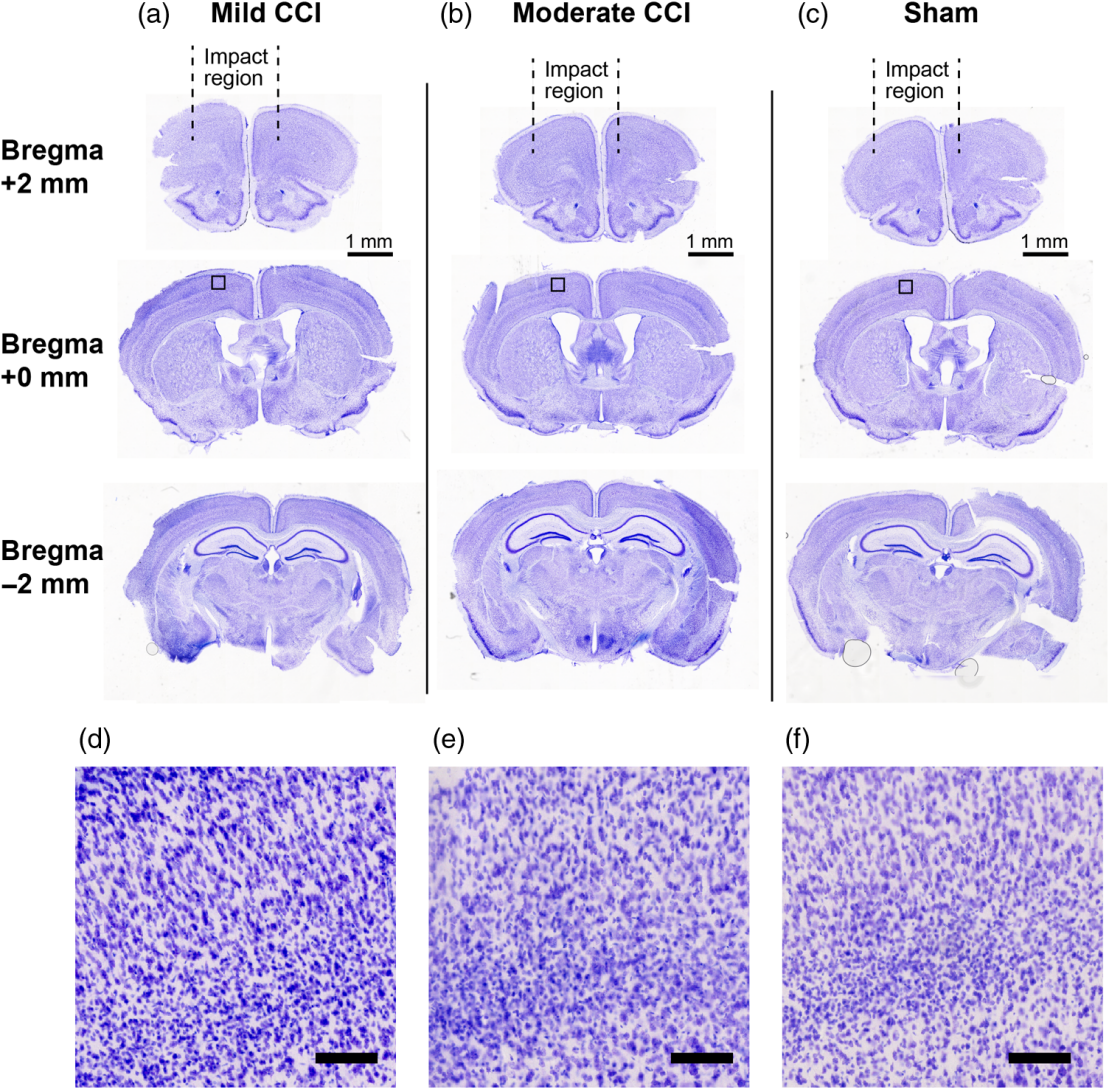
CV staining reveals negligible macroscopic anatomical damage 24 h after CCI. Columns (a) and (b) depict CV-stained coronal brain sections from mice that received “mild” and “moderate” CCI treatment, respectively, and (c) depicts CV-stained sections from sham experiments. Here, “mild” CCI corresponds to the parameter settings employed for all of the functional data in this study (velocity: 4  m/s; dwell time: 100 ms; impact depth: 0.6 mm), while “moderate” corresponds to an increased impact depth (1.1 mm), with other parameters remaining the same. Slices from each condition are shown at locations on the AP axis corresponding to +2  mm, 0 mm, and −2  mm relative to bregma. Square subregions that are indicated in the slices at bregma +0  mm (middle row) are shown on an expanded scale (imaged at 40×, scale bar=100  μm) in (d)–(f).

**Fig. 8:**
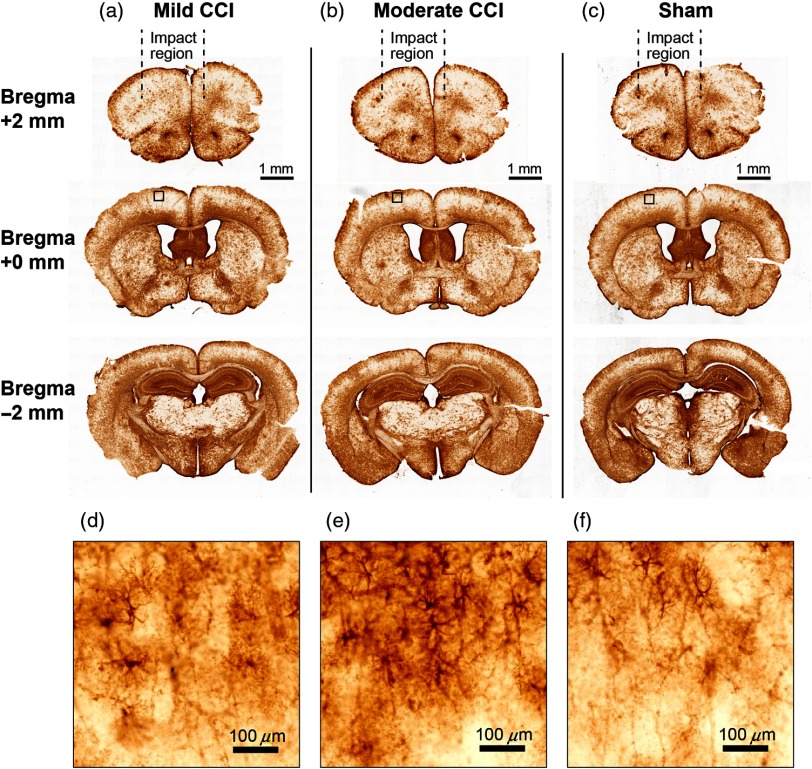
GFAP immunoreactivity 24 h after CCI indicates mild astrogliosis, but no macroscopic damage. Following the layout of Fig. 7, columns (a) and (b) depict GFAP immunoreactivity in coronal slices from mice that received mild and moderate CCI treatment, respectively, and (c) depicts results from sham experiments. Definitions of mild and moderate are as described in the caption for Fig. 7. Mild CCI corresponds to the parameter settings employed for all of the functional data in this study. The small notches on the ventrolateral corners of the brain slices indicate the hemisphere contralateral to the central axis of the impactor tip. Subregions that are indicated in the slices at bregma +0  mm (middle row) are shown on an expanded scale in (d)–(f).

To assess the repercussions of our implementation of cortical impact on the brain, we explored both macroscopic, tissue-level effects as well as GFAP expression in brains of animals that had been exposed to CCI (Figs. 7 and 8, respectively). We assessed effects at locations (1) close to the site of impact, which was anterior to the site of DCS and SSEP measurements, (2) at the same AP location as the optical and electrophysiological measurements, and (3) at a location distal (posterior) to the site of impact.

**Fig. 9:**
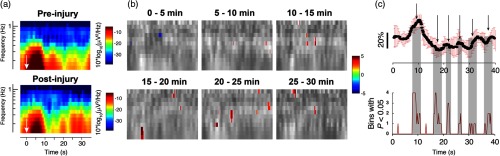
Time-frequency analysis reveals significant changes in spectrogram after injury. (a) Representative time-frequency spectrograms from a single animal showing the alterations in spectrotemporal content following CCI. The white arrows indicate the time point at which forelimb stimulation was applied. (b) Spectrotemporal changes over time, averaged over all animals. Each image depicts, in grayscale, the difference in spectrotemporal content via subtraction of the preinjury spectrogram (darker shades indicate decreases, brighter shades indicate increases). Superimposed in color on the grayscale difference spectrogram are points that, when analyzed over all experiments, demonstrated a difference from the preinjury values with P<0.05 using a two-tailed student’s t-test. The colors are defined as in the colorbar of (a), and note that nearly all of the spectrotemporal power changes are focal increases. (c) Comparison of a representative postinjury ΔCBF waveform (here, at a latency of 25 min following injury) with a plot showing the sum, over all experiments, of pixels showing alterations from baseline with P<0.05. Note that the significantly changed spectrotemporal features lined up with the cycles of apparent “oscillations” following injury. The gray shading projected onto the postinjury ΔCBF waveform (a representative plot) highlights temporal periods that contained statistically significant alterations in spectral power, assessed over all experiments (n=10).

**Fig. 10:**
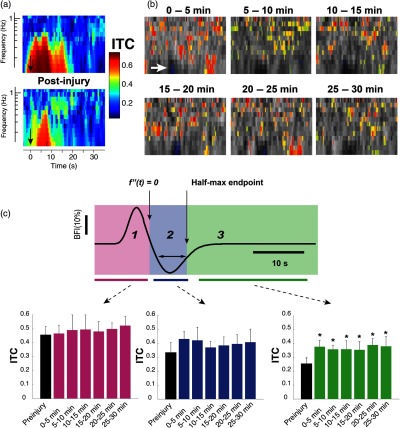
ITC alterations following controlled cortical injury. (a) Representative spectrograms depicting the absolute magnitude of ITC before (top) and after (bottom) injury. These particular spectrograms are derived from the same animal as in Fig. 9(a). The color definitions are as indicated in the ITC colorbar; a value of 1 represents perfect, in-phase spectral responses. While the depicted values are real values, they represent the absolute magnitude of complex values that originally contain phase information. The black arrows indicate the time point at which forelimb stimulation was applied. (b) A series of ITC spectrograms, averaged over all animals, showing features that differ significantly from preinjury with P<0.05 obtained from a two-tailed student’s t-test. As in Fig. 9(b), the statistically significant pixels, which are color-coded according to the colorbar of (a), are superimposed on a grayscale spectrogram representing the postinjury ITC spectrogram from which preinjury spectrogram values have been subtracted. While most of the changes represent increases in ITC at time points following the initial ΔCBF waveform peak and undershoot, there was a significant decrease in ITC in the region of the undershoot itself, roughly the area of frequencies below 0.4 Hz between 12- and 16-s poststimulus (indicated by the white arrow in the top left superimposed image). (c) Regional assessment of ITC changes following injury. A sample ΔCBF waveform fit divided into three regions for assessing changes in ITC at particular phases of the functional blood flow response is shown on top. The specific regions were defined as (1) an initial “rise” extending from the time directly after sensory stimulation cessation up through the point where the second derivative of the ΔCBF fit passes zero; (2) the undershoot period, extending from the end of (1) through the latest point of intersection of the ΔCBF fit with the undershoot half-maximum (or minimum, here) line; (3) the period of time following the main two phases of the ΔCBF waveform and extending until the subsequent stimulus period. Asterisks in the right-most bar chart on the bottom indicate values that differ from baseline, preinjury values with P<0.05 according to a two-tailed Student’s t-test.

**Fig. 11:**
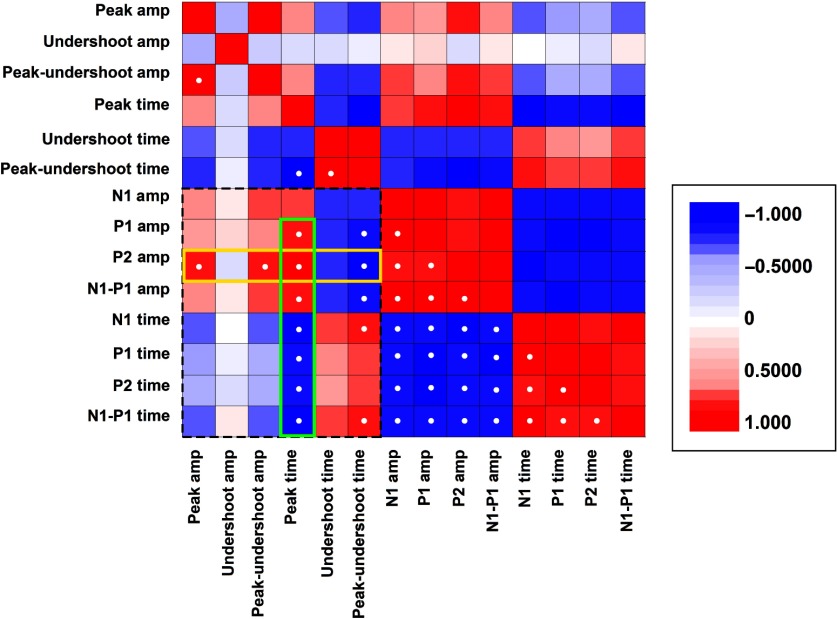
Correlation matrix comparing the post-CCI recovery trends for the prominent features of the CBF and SSEP responses. Each matrix element represents the Pearson’s correlation coefficient obtained by comparing the post-CCI time course (as in Figs. 4 and 5) of the major peaks and latencies. A correlation coefficient value of 1 represents identical waveforms, −1 indicates that the two traces are perfectly anticorrelated. The matrix is symmetrical about the diagonal. Correlation coefficients with an absolute value higher than 0.8 are indicated with white dots. The dotted rectangular perimeter delimits the matrix region representing correlation between the two modalities, optical and electrophysiological. The yellow perimeter highlights the high correlation between the P2 amplitude and postinjury hemodynamics, whereas the green perimeter highlights the fact that the ΔCBF peak latency is highly correlated with all aspects of SSEPs.

Figure 7 shows CV Nissl staining of brain sections from animals that had been exposed to CCI. Figure 7(a) shows CV-stained slices from an animal subjected to the same CCI parameters used during acquisition of all functional data (i.e., Figs. 3–6 and 9–11); we termed these settings “mild” for the sake of comparison with the histological results obtained following a slightly more intense impact (i.e., impact depth of 1.1 mm versus 0.8), which we termed “moderate.” Compared with sham animals, CV staining revealed no evidence of significant alterations in tissue morphology, such as persisting edema, major hematoma, or direct lesions resulting from the CCI settings used throughout this study (two animals per condition). GFAP immunoreactivity (Fig. 8), however, revealed a proliferation of reactive astrocytes biased toward the hemisphere that was exposed to a greater area of the impactor tip (which was 5 mm in diameter). This astrogliosis effect was evident at the site on the AP axis where functional measurements were centered and was more pronounced at moderate CCI (impact depth 1.1 mm) compared with mild (0.8 mm). Although GFAP immunoreactivity appeared to be more globally intense at the most distal region assessed (AP −2  mm), this is consistent with baseline expression patterns of GFAP,^36^ and there was no apparent difference relative to the sham condition.

### 3.4 Spectrotemporal Analysis the Hemodynamic Response Function Following TBI

Modulation of prominent aspects of the hemodynamic response such as peak amplitude and latency are straightforward to quantify because the waveforms can fit well to a conventional HRF model (as described in Sec. 2). However, injury also induced new temporal features into the CBF response. For example, in the ∼30  s following each evoked peak in CBF, there emerged slow oscillations in flow at a frequency of roughly 0.1 to 0.3 Hz. Such low-frequency oscillations have been reported both as spontaneous fluctuations in ongoing flow^37^^–^^39^ as well as following functional activation.^40^ These emerging oscillations introduce additional waveform peaks that cannot be effectively fit using the standard, two-gamma function model. As an alternative approach for quantifying these new components of the CBF response, we performed time-frequency spectral analysis to explore and more directly visualize these effects. While not typically applied to evoked responses, such spectral analysis has been used to explore dynamic, low-frequency aspects of ongoing cerebral autoregulation^41^ and provides an approach for characterizing the putative oscillatory behavior. As shown in Fig. 9, injury elicited changes in spectral power throughout the duration of the major peaks (increase and undershoot) of the CBF response, as well as in the interim between stimulus sets. Although the apparent spectrotemporal changes following injury resemble the development of temporally separated “notches” in the spectral power for frequencies below 1 Hz, we explored whether these changes were significant across animals by assessing the patterns of spectrotemporal differences from preinjury values [Fig. 9(b)]. The regions of statistically significant spectrotemporal changes in power are relatively sparse; however, when summed over frequency and for all experiments, significant changes coincided with individual peaks in the “oscillatory” region, indicating a degree of phase locking [Fig. 9(c)].

To quantify this apparent coherence effect, we explored the ITC (Fig. 10), which can reveal spectrotemporal phase locking and variability among evoked responses.^35^ Similar to the results of spectral power analysis shown in Fig. 9, injury qualitatively changed ITC most prominently for regions following the initial CBF peak. A large fraction of the alterations in ITC were increases, most prominently in the period after the CBF undershoot and for frequencies above 0.4 Hz. The one region that displayed a significant decrease in ITC was that corresponding to the spectrotemporal representation of the CBF undershoot, indicating greater variability of that portion of the HRF after injury. Although significant spectrotemporal increases in ITC (P<0.05 via two-tailed student’s t-test) were apparent throughout the time course of the CBF response, we investigated the temporal distribution of ITC alterations [Fig. 10(c)] by analyzing flow as a series of three “regions” defined by physiologically relevant landmarks. When summed over frequencies, the period following the ΔCBF undershoot (region three) demonstrated significant alterations from baseline.

### 3.5 Correlations in the Dynamics of Cerebral Blood Flow and Electrophysiological Responses Following TBI

Given the diversity of signals that were sensitive to injury, we sought to merge the findings by exploring correlations among their recovery kinetics. The matrix in Fig. 11 presents correlation coefficients comparing the post-CCI quantitative dynamics shown in Figs. 4 and 5. Overall, SSEP peak amplitudes and latencies were highly intercorrelated. In comparison, the recovery kinetics of the CBF components was relatively weakly intercorrelated. Among the cross-correlation terms indicative of coupling between hemodynamic and neural activity, we identified two major trends that are highlighted in Fig. 11. Notably, of all time-domain electrophysiological features that we explored, the amplitude of the CBF peak was most significantly correlated with the amplitude of P2 (z-score>3, based on the SD of the distribution of Pearson’s correlation coefficients). Additionally, while P2 represented the strongest cross-modal correlation in terms of amplitude, the latency of the initial ΔCBF peak was highly correlated with all aspects of the SSEP peaks.

## 4 Discussion

A major repercussion of traumatic injury in the brain is an ensuing mismatch between oxygen supply and neural metabolic demand.^42^^,^^43^ Changes in CBF and local tissue oxygenation dynamics ultimately reflect an interplay between CBF and changes in the cerebral metabolic rate of oxygen (${\mathrm{CMRO}}_{2}$), both of which are susceptible to alteration following acute injury. The primary mechanical injury, for example, can alter the vascular network that delivers oxygen to the parenchyma and, simultaneously, can alter neural activity and metabolic rate through a wide spectrum of mechanisms, including mechanical shearing^44^ or excitotoxicity due to damaged glia.^45^ Our concomitant measurements of CBF and electrical activity provide a picture of the repercussions of injury on both “supply and demand” aspects of the networks underlying sensation. While it is possible for injury to globally affect brain oxygenation (and, indeed, systemic physiology) as a result of compression of cardiorespiratory brainstem nuclei, our histological assessment does not find evidence of this scenario, and the changes in physiological signals likely reflect more subtle, transient perturbations.

While the general trends in SSEP alterations following injury are consistent with previous work in animal models,^23^^,^^25^^,^^26^^,^^46^ in the present study, we were able to identify time-domain alterations in the SSEP in finer detail. The most prominent SSEP peaks—N1, P1, and N2—exhibited differing alterations after injury, some of which are consistent with the known anatomical correlates of time-domain waveform. For instance, considering that the site of primary injury in CCI is the cortical gray matter,^47^ it is not entirely surprising that P1 (∼13-ms onset delay preinjury) exhibited the mildest decrease following injury given the fact that this component of the SSEP reflects subcortical activity related to thalamocortical relay.^48^ Likewise, the high sensitivity of P2 likely reflects the fact that its underlying generators are derived from cortico-cortical processing, which is sensitive to lateral cortical perturbations. For example, modulating cortical activity with topical application of pharmacological agents generally affects N1, but largely spares P1.^49^ Consistent with this, in our previous work using a high-intensity focused ultrasound model for explosive blast, the mechanical insult penetrated deeper beneath the surface of the cortex and P1 was more significantly altered.^23^ The high correlation between hemodynamics and P2 may also reflect an intrinsic, disproportionately large influence of superficial cortical injury on electrical activity and hemodynamics within the cortical microvasculature. The superficial layers of the cortex feature extensive lateral networking with other cortical areas;^50^ thus, local disruption due to impact injury would likely have diffuse and far-reaching repercussions.

Within the temporal span of individual hemodynamic responses, the major features of the preinjury ΔCBF that we observed—including both an initial peak and subsequent undershoot—have been observed in other DCS studies^51^^,^^52^ as well as studies using laser speckle imaging.^17^ While evidence of a significant undershoot is lacking in laser Doppler measurements, a clear inflection point between the CBF peak and return to baseline has been observed.^53^ A significant undershoot has also been reported in some fMRI studies, measured through arterial spin labeling,^54^^–^^56^ but not all.^57^ A major contributing factor underlying discrepancies in CBF results is likely the anatomical specificity of the measurement modality. For example, DCS measurements and speckle flow imaging are highly sensitive to flow in smaller vessels, whereas laser Doppler, which yields a minimal undershoot, is biased toward signals in large vessels, which generally exhibit smaller fractional changes in flow during functional activation.

The initial CBF peak reflects the influx of blood flow recruited by local changes in neural activity,^58^ and, on the timescale of days, its amplitude has been found to be reduced following traumatic injury^59^ and stroke.^60^ To our knowledge, however, the impact of injury on sensory-evoked CBF, both in terms of amplitude and temporal waveform, has not been explored within the first moments following TBI. It remains to be seen whether our observed acute dynamics, which depict significant recovery of some aspects of the sensory-evoked ΔCBF, continuously evolve into the previously reported long-term deficits. In terms of injury-induced alterations in peak CBF timing, the same prior investigations reported a delayed flow onset that is observable on the timescale of days following injury. Our observation of a reduced onset latency is innovative; however, it may be unique to the immediate aftermath of acute injury. In general, the fact that most aspects of the ΔCBF response and SSEPs substantially recover within a relatively short period of time is remarkable given the known chronic effects that TBI exerts on baseline hemodynamics, notably, changes in cerebrovascular autoregulation.^61^^,^^62^ The findings concur with previous reports that functional CBF is independent of baseline values;^53^^,^^63^^,^^64^ however, our results indicate that this independence extends to injury-induced global hemodynamic changes.

It has long been assumed that the sensory-evoked hemodynamic response undershoot is primarily a vascular phenomenon, representing a poststimulus period of increased cerebral blood volume even after CBF and ${\mathrm{CMRO}}_{2}$ have returned to baseline.^65^ However, more recent work strongly indicates that the poststimulus undershoot is highly dependent on neural activity.^56^ Our results depict the postinjury undershoot as chronically attenuated and more variable. Similarly, SSEP components also displayed a marked increase in variability in both time and frequency domain. These observations are, therefore, consistent with the hypothesis that neural activity plays a significant role in shaping the undershoot. However, the fact that the variability and amplitude of the undershoot does not recover as fully as other CBF waveform components even after SSEP attributes have returned to baseline status suggests that the prolonged undershoot alterations may not be primarily shaped by neural activity. Future studies involving systematic variation of stimulus parameters may be able to more directly probe this hypothesis.

Using a variety of techniques, low-frequency (<0.5  Hz) CBF oscillations similar to the ones we detected following injury (Figs. 9 and 10) have been previously observed, both spontaneously and following stimulus-evoked CBF, in the form of vasomotion.^37^^,^^40^^,^^66^^,^^67^ While the physiological basis of these oscillations is still currently unclear, multiple mechanisms have been suggested, including emergent properties of molecular signaling pathways^68^ or more fundamental fluid mechanical principles.^69^^,^^70^ Such fluid mechanical models derive an oscillatory component based on inclusion of an inertial term, owing to blood volume in larger, more distal venules and arterioles.^71^ A simplistic, general interpretation of the results is that the emergence of postundershoot oscillations in CBF reflects perturbation of an intrinsically oscillatory system, regardless of whether the affected components are mechanical or phenomenological (e.g., related to molecular signaling).

In emergency medicine, there is a well-known principle of the “golden hour,” the period of time following a traumatic injury within which medical intervention can have a maximal impact on patient outcome.^72^ Although the precise duration is debated,^73^^,^^74^ timely transport to a hospital is essential. Our experimental investigation focused on disruptions in neurovascular coupling that occurs within this relatively short time window, and we identified several metrics that were profoundly altered by TBI. It is, however, both possible and likely that at later time points, the trends we observed will change or reverse. The rapid energy depletion associated with disrupted CBF and mechanical shearing of neurons dramatically increases extracellular $K^{+}$ and glutamate concentrations.^75^^,^^76^ In turn, resulting excitotoxicity triggers a variety of rapidly evolving adverse events through the “ischemic cascade.” For example, initial cytotoxic edema is followed by the onset of vasogenic edema within the first few hours.^77^^,^^78^ These various sequelae will likely be reflected in the optical and electrophysiological signals. Future experimental work focusing on the longer-term evolution of neurovascular coupling following CCI could provide a fuller physiological context for the initial effects that we characterized in this present study. On a technical level, focusing on physiological signals during the early timeframe of injury provided a way to mitigate potential confounds owing to anesthesia pharmacodynamics, which could be affected by injury.

A general challenge for parsing the bulk signals obtained through diffuse optical neuromonitoring techniques is that the spatial resolution is limited because the detected signals include light that has traversed a large span of optical paths in tissue.^79^ In the case of DCS, although the signal origin is putatively weighted toward microvasculature, the fidelity with which the bulk signal aligns with a microscale portrait of blood flow in the layers of the cerebral cortex remains to be quantified. Future work in animal models of TBI that supplements DCS measurements with high-resolution imaging of activity in microvasculature, such as optical coherence angiography^80^^,^^81^ or two-photon microscopy,^82^ may better inform interpretation.

## 5 Conclusion

While significant progress has been made toward understanding the repercussions and mechanisms of mTBI at the cellular level using invasive, high-resolution techniques, biomarkers for rapidly assessing human subjects in active settings outside the clinic are constrained to signals that can be obtained with noninvasive technology. Such measurement modalities, however, are limited in their ability to spatially resolve signals and biomarkers observed in preclinical work. As we have demonstrated, functional neurovascular coupling is a property that can be measured with low-resolution techniques and contains information about microscale physiological function. This information can be extracted from variable, high-noise physiological recordings because they are temporally synchronized with external stimuli that can be delivered in a controlled manner. The event-related optical and electrophysiological changes that we have discovered, albeit correlative, are significantly indicative of TBI in an animal model. Notably, the dynamic alterations we observed in stimulus-evoked blood flow and electrophysiological responses were not associated with significant tissue damage; rather, our histological examination revealed only a small increase in astrogliosis associated with CCI. We expect that these functional indicators will ultimately add informative dimensions to current multimodal approaches for diagnosing mTBI in human subjects, given the heterogeneity of baseline values across populations.
